# The role of potential probiotic strains *Lactobacillus reuteri* in various intestinal diseases: New roles for an old player

**DOI:** 10.3389/fmicb.2023.1095555

**Published:** 2023-02-02

**Authors:** Zihan Yu, Jihua Chen, Yaxin Liu, Qingguo Meng, Hang Liu, Qinyan Yao, Wenxuan Song, Xiangfeng Ren, Xin Chen

**Affiliations:** ^1^Department of Gastroenterology and Hepatology, Tianjin Medical University General Hospital, Tianjin, China; ^2^Tianjin Institute of Digestive Disease, Tianjin Medical University General Hospital, Tianjin, China

**Keywords:** *Lactobacillus reuteri*, intestinal diseases, gut microbiota, inflammatory bowel disease, colorectal cancer

## Abstract

*Lactobacillus reuteri* (*L. reuteri)*, a type of *Lactobacillus* spp., is a gut symbiont that can colonize many mammals. Since it was first isolated in 1962, a multitude of research has been conducted to investigate its function and unique role in different diseases as an essential probiotic. Among these, the basic functions, beneficial effects, and underlying mechanisms of *L. reuteri* have been noticed and understood profoundly in intestinal diseases. The origins of *L. reuteri* strains are diverse, with humans, rats, and piglets being the most common. With numerous *L. reuteri* strains playing significant roles in different intestinal diseases, DSM 17938 is the most widely used in humans, especially in children. The mechanisms by which *L. reuteri* improves intestinal disorders include protecting the gut barrier, suppressing inflammation and the immune response, regulating the gut microbiota and its metabolism, and inhibiting oxidative stress. While a growing body of studies focused on *L. reuteri*, there are still many unknowns concerning its curative effects, clinical safety, and precise mechanisms. In this review, we initially interpreted the basic functions of *L. reuteri* and its related metabolites. Then, we comprehensively summarized its functions in different intestinal diseases, including inflammatory bowel disease, colorectal cancer, infection-associated bowel diseases, and pediatric intestinal disorders. We also highlighted some important molecules in relation to the underlying mechanisms. In conclusion, *L. reuteri* has the potential to exert a beneficial impact on intestinal diseases, which should be further explored to obtain better clinical application and therapeutic effects.

## Introduction

According to the World Health Organization, probiotics are defined as “live microorganisms which, when administered in adequate amounts, confer a health benefit on the host” (Mu et al., 2018). These probiotics, that have already been proven to exert a beneficial impact, include *Saccharomyces boulardii, Lactobacillus* spp., *Bifidobacterium* spp., *Propionibacterium* spp., *Streptococcus* spp., *Bacillus* spp., *Enterococcus* spp., and some specific strains of *Escherichia colietc*, in which *Lactobacillus* spp. is the most widely used in human nutrition (Kechagia et al., 2013; Markowiak and Slizewska, 2017).

Recently, numerous bodies of research demonstrated that probiotics are beneficial for various diseases, such as intestinal disorders, respiratory tract infections, vaginal diseases, and so on (Markowiak and Slizewska, 2017; Nami et al., 2018). With the development of the food and drug industries and the innovation of technology, an increasing number of emerging probiotic strains were developed and applied to different fields, including natural food preservatives, nutraceuticals, and so on (Nami et al., 2015, 2018). Some novel technologies can also enhance probiotic products' quality and sensory characteristics, which can contribute to the extensive application of probiotics (Kiani et al., 2021a,b).

Safety is also an essential issue during the process of investigating probiotics. An ocean of evidence indicated that the use of probiotics can cause some risks regarding systemic infections, deleterious metabolic activities, excessive immune stimulation in susceptible individuals, gastrointestinal side effects, and so on (Doron and Snydman, 2015). Some probiotic microorganisms can even transfer resistance genes to protect against antibiotics, which may be responsible for the development of the antibiotic resistance crisis (Daniali et al., 2020). Taken together, the application of probiotics is a double-edged sword. Before probiotics are used, we still need to carry out enough clinical trials and animal experiments to assess their benefits and harms.

*Lactobacillus* spp., which can be found in various food products, is one of the most widely used probiotics, and includes *Lactobacillus acidophilus, Lactobacillus rhamnosus, Lactobacillus bulgaricus, Lactobacillus casei*, and *Lactobacillus reuteri* (*L. reuteri*) (Giraffa et al., 2010). *Lactobacillus reuteri* is a gut symbiont mainly colonized in the intestines of humans, rodents, pigs, and chickens (Oh et al., 2010; Walter et al., 2011; Rattanaprasert et al., 2019). Since first isolated in 1962, there have been a great number of studies conducted on *L. reuteri* to explore its functions and concrete mechanisms in different diseases, which cover gastrointestinal diseases, hypercholesterolemia, skin infection, allergic asthma, periodontitis, hand, foot, and mouth disease (HFMD), and so on (Prince et al., 2012; Ang et al., 2016; Giudice et al., 2016; Mu et al., 2018; Tachi et al., 2018; Theodoro et al., 2019; Wang et al., 2022). Among these, there is an increasingly prevalent trend that the investigations of *L. reuteri* in terms of intestinal diseases are becoming far and away the best area, which mainly concentrates on inflammatory bowel disease (IBD), colorectal cancer (CRC), children's functional bowel diseases, and so on.

Intestinal diseases, especially IBD, have become an increasing burden on the global healthcare system and society. The prevalence of IBD is expected to increase to 790 per 100,000 in 2025 (Morales et al., 2017). Of note, there will be over 1.5 million cases of IBD in China by 2025 (Kaplan, 2015). The increased use of biological therapies and the aging population will bring new challenges and complexities to public healthcare and society's economy (Kaplan, 2015). Moreover, IBD is one of the leading causes of CRC, which is the third most common cancer globally (Weitz et al., 2005; Keller et al., 2019). Although the incidence of CRC has decreased overall, it has been estimated that the incidence rates for colon and rectal cancers may increase by 90.0 and 124.2%, respectively, for patients between the ages of 20 and 34 by 2030 (Bailey et al., 2015). This phenomenon may be attributed to the fact that the pediatric overall prevalence of IBD has increased sharply (Ye et al., 2020). IBD, including ulcerative colitis (UC), and Crohn's disease (CD), is a type of chronic relapsing-remitting disease characterized by intestinal inflammation (Guo X. et al., 2021). Conventional treatment approaches include mesalazine, glucocorticoids, immunosuppressors, and so on. However, considering multiple investigations on gut microbiota and IBD (David et al., 2014; Jost et al., 2014; Thaiss et al., 2014; Ananthakrishnan, 2015), probiotics are expected to become an effective treatment for this disease. Notably, there is an abundance of studies on the fact that *L. reuteri* exhibits the following beneficial capacities: anti-inflammation, immune regulation, gut micro-ecology balance, gut barrier protection, metabolic control, and so on (Mu et al., 2018).

In this study, we comprehensively reviewed the literature concerning *L. reuteri* and its metabolites in the pathogenesis of several common intestinal diseases, which are illustrated in a separate section for a clearer understanding. Some basic introductions and future perspectives are also discussed in this review (Tables 1–3).

**Table 1:** Therapeutic efficacy and potential mechanisms of *Lactobacillus reuteri* strains in various intestinal diseases in cell models.

***Lactobacillus reuteri* strain**	**References**	**Experimental model/participant**	**Disease**	**Effect/outcome**	**Mechanism of action**
I5007	Marcinkiewicz et al. (2007)	LPS-induced human colon cell line HT-29 cells	IBD	Reduced pro-inflammatory cytokines levels	Inhibition of NF-κB pathway
NK33	Mackos et al. (2016)	LPS-induced BV2 and SH-SY5Y cells	Anxiety/ depression and colitis	Inhibited IL-6 expression; increased LPS-suppressed CREB phosphorylation as well as BDNF expression	Inhibition of NF-κB pathway
MG5346	Fong et al. (2020)	Human colorectal carcinoma RKO cells	CRC	Induced cell apoptosis	Activation of Caspase-9-Dependent Apoptosis pathway
LFCA-encoding *L. reuteri* CO21 (LR-LFCA)	Gao et al. (2017)	LPS-induced IPEC-J2 cells	Infectious bowel disease	Reduced oxidative stress and inflammatory factors	Activation of the NRF2/HO-1 pathway; inhibition of the NF-κB pathway
L26 Biocenol (CCM 8616)	Zhang et al. (2018)	ETEC-induced IPEC-1 cells	Infectious bowel disease	Attenuated overexpression of the gene and suppressed inflammatory responses	The underlying mechanisms remain unclear
ATCC PTA 6475	Bell et al. (2022)	ETEC-induced IPEC-J2 cells	Infectious bowel disease	Protected the mucosal barrier and reduced inflammatory factors	The underlying mechanisms remain unclear
DSM 17938	Bell et al. (2022)	ETEC-induced IPEC-J2 cells	Infectious bowel disease	Protected the mucosal barrier and reduced inflammatory factors	The underlying mechanisms remain unclear
1563F	Bell et al. (2022)	ETEC-induced IPEC-J2 cells	Infectious bowel disease	Protected the mucosal barrier and reduced inflammatory factors	The underlying mechanisms remain unclear
LR1	Martín-Cabrejas et al. (2017)	ETEC-induced IPEC-1 cells	Infectious bowel disease	Decreased the adhesion and invasion of the coliform in IPEC-1 cells; increased transcript abundance and protein contents of TJ proteins ZO-1 and occludin and enhanced epithelial barrier	Activation of MLCK pathway
ATCC PTA 6475	Watschinger and Moschen (2022)	EPEC-induced human colon carcinoma HT-29 (ATCC HTB-38) and LS174T cells (ATCC CL-188)	Infectious bowel disease	Adherence of *L. reuteri* to HT-29 cells was strain-specific; inhibited EPEC binding to HT-29 but not LS174T cells; decreased EPEC adherence to small intestinal biopsy epithelium	The underlying mechanisms remain unclear
ATCC 53608	Watschinger and Moschen (2022)	EPEC-induced human colon carcinoma HT-29 (ATCC HTB-38) and LS174T cells (ATCC CL-188)	Infectious bowel disease	Adherence of *L. reuteri* to HT-29 cells was strain-specific; inhibited EPEC binding to HT-29 but not LS174T cells; decreased EPEC adherence to small intestinal biopsy epithelium	The underlying mechanisms remain unclear
ATCC 55730	Karimi et al. (2018)	Peritoneal macrophages	Infectious bowel disease	Activated macrophages and enhanced the ability of macrophages to phagocytose and to kill intracellular *Salmonella* Typhimurium; increased the secretion of NO in macrophages and enhanced the anti-inflammatory effect	The underlying mechanisms remain unclear

IBD, inflammatory bowel disease; NF-κB, nuclearfactor-κB; LPS, lipopolysaccharide; BDNF, brain-derived neurotrophic factor; CREB, cAMP-response element binding protein; NRF2, nuclear factor erythroid 2-related factor 2; HO-1, heme oxygenase 1; TJ, tight junction; MLCK, myosin light-chain kinase; NO, nitric oxide; IPEC-1, Intestinal porcine epithelial cell; IPEC-J2, porcine jejunal epithelial cell; TJ, tight junction; ZO-1, zonula occluden-1; MLCK, myosin light-chain kinase.

**Table 2:** Therapeutic efficacy and potential mechanisms of *Lactobacillus reuteri* strains in various intestinal diseases in animal models.

***Lactobacillus reuteri* strain**	**References**	**Experimental model/participant**	**Disease**	**Effect/outcome**	**Mechanism of action**
R28	Xu et al. (2021)	PEG + DSS-induced C57BL/6 mice	IBD	Reduced diarrhea; reduced pro-inflammatory cytokines; enhanced the intestinal barrier	The underlying mechanisms remain unclear
ATCC PTA 4659	Guarner et al. (2002)	DSS-induced C57BL/6J mice	IBD	Reduced pro-inflammatory cytokines; prevented the CD11b^+^Ly6G^+^ neutrophil recruitment; reduced the CD11b^+^CD11c^+^ DCs; Foxp3^+^CD4^+^ t cells decreased in MLNs	Upregulated HSPs family
5454	Shin and Kim (2018)	TNBS-induced C57BL/6J and BALB/c ByJ mice	IBD	Improved colitis; induced tolerogenic DCs and triggered IL-22 secretion	Induction of Tregs
ATTC PTA 6475	Guo F. et al. (2021)	TNBS-induced BALB/c mice	IBD	Suppressed inflammation; promoted DCs maturation, stimulated IL-10 production	The underlying mechanisms remain unclear
I5007	Marcinkiewicz et al. (2007)	DSS-induced C57BL/6 mice	IBD	Reduced weight loss, colon length shortening, and histopathological damage; restored the mucus layer; reduced pro-inflammatory cytokines levels; altered colonic microbiota and metabolic structural and functional composition	Inhibition of NF-κB pathway; stimulated the expression of MUC2, increased the number of goblet cells; the enrichment of KEGG pathways, such as ABC transporters and carbohydrate metabolism-related pathways
ATCC PTA 6475	Liu et al. (2022)	DSS-induced C57BL/6 mice	IBD	Reduced weight loss; ameliorated the immunopathology and inflammatory status	Reduction of ILC3s
BR11	Wang G. et al. (2020)	DSS-induced Sprague–Dawley rats	IBD	Improvement in crypt area;	The underlying mechanisms remain unclear
23272	Khalil et al. (2016)	*Citrobacter rodentium*-induced C57BL/6 mice and CCL2^−/−^ mice	IBD	Reduced the stressor effects and histopathological damage	Down-regulation of the chemokine CCL2
Clade II strain 6475	Weger and Sandi (2018)	TNBS-induced BALB/c mice	IBD	Diminished weight loss, colonic injury, serum amyloid A (SAA) protein concentrations, and reduced uptake of [^18^F]FDG	Activation of hdc/l-histidine/histamine/H2R pathway
F-9-35	Jang et al. (2018b)	DSS-induced ICR mice	IBD	Had less inflammatory Phenotype; reduced myeloperoxidase activity, and lower expression of proinflammatory genes (TNF-α, COX-2 and IL-6); alleviation of microbiota dysregulation	The underlying mechanisms remain unclear
NK33	Mackos et al. (2016)	Immobilization stress (IS)-induced C57BL/6 mice	Anxiety/ depression and colitis	Alleviated the occurrence and development of anxiety/depression and colitis; suppressed infiltration of Iba1+ and LPS^+^/CD11b^+^ cells (activated microglia) into the hippocampus, and corticosterone, IL-6, and LPS levels in the blood	Inhibition of NF-κB pathway; increase of BDNF expression and CREB phosphorylation
ATCC PTA 6475	He et al. (2022)	AOM-induced BALB/c mice, hdc^−/−^ BALB/c mice	CRC	Reduced the number and size of colon tumors	Activation of hdc/histamine/H2R pathway
MG5346	Fong et al. (2020)	Human colorectal cancer xenografts in BALB/c nude mice	CRC	Inhibited tumor growth	Activation of Caspase-9-Dependent Apoptosis pathway
LFCA-encoding *L. reuteri* CO21 (LR-LFCA)	Gao et al. (2017)	ETEC-induced piglets	Infectious bowel disease	Attenuated the weight loss and diarrhea incidence; improved the intestinal morphology, intestinal epithelial barrier and increased the expression of intestinal tight junction proteins; improved the gut microbiota; modulated gut immune responses; reduced oxidative stress and inflammatory factors	Activation of the NRF2/HO-1 pathway; inhibition of the NF-κB pathway
HCM2	Asare et al. (2020)	ETEC-induced ICR mice	Infectious bowel disease	Inhibited the growth of ETEC and its ability to adhere to intestinal epithelial cells; preserved intestinal morphology; stabilized the gut microbiota	The underlying mechanisms remain unclear
TMW1.656	Zhang et al. (2020)	Weanling piglets	Infectious bowel disease	Reduced the copy numbers of genes for *E. coli* and the heat-stable enterotoxin in feces, reduced the level of colonization of weaning piglets with ETEC	The underlying mechanisms remain unclear
LTH5794	Zhang et al. (2020)	Weanling piglets	Infectious bowel disease	Reduced the copy numbers of genes for *E. coli* and the heat-stable enterotoxin in feces; reduced the level of colonization of weaning piglets with ETEC	The underlying mechanisms remain unclear
Lb11	Xie et al. (2021)	Eggs and chickens	Infectious bowel disease	Inhibited the growth of *Salmonella* enteritidis	Reduction of the AcrAB-TolC efflux pump genes, outer membrane protein genes and antibiotic resistance genes
KUB-AC5	Tkáčiková et al. (2020)	*Salmonella* enteritidis S003-induced broiler chickens (Ross 308)	Infectious bowel disease	Maintained the stabilization of gut microbiome; enhanced Lactobacillaceae levels in both the ileum and caecum and suppressed Enterobacteriaceae levels	The underlying mechanisms remain unclear
ATCC 55730	Karimi et al. (2018)	*Salmonella* Typhimurium-induced C57BL/6 mice	Infectious bowel disease	Reduced weight loss; prolonged the survival of mice; inhibited the dissemination of S. typhimurium from the abdominal cavity to the spleen and liver	The underlying mechanisms remain unclear
CCM 8617	Yi et al. (2018a)	*Salmonella* Typhimurium CCM 7205NAL-induced BALB/c mice	Infectious bowel disease	Reduced the growth of Salm. Typhimurium; alleviated the negative impact of Salm. Typhimurium; the liver showed marked reduction of overall inflammation, hepatocyte necrosis and size of typhoid nodules	The underlying mechanisms remain unclear
SLZX19-12	Yang et al. (2015)	*Salmonella* Typhimurium SL1344-induced C57BL/6J mice	Infectious bowel disease	Lower loads of *Salmonella* in visceral organs, less colonic inflammation, and higher barrier integrity; more stable microbiota structure of the colon, in which the abundance of Alloprevotella was greatly enhanced	The underlying mechanisms remain unclear
ATCC 23272	Walsham et al. (2016)	HRV-induced gnotobiotic pigs	Infectious bowel disease	Enhanced Th1 and Th2 cytokine responses to HRV infection; regulated TGF-β production to maintain immune homeostasis	The underlying mechanisms remain unclear
L26 Biocenol^TM^	Eaton et al. (2011)	PCV2-induced germ-free Balb/c mice	Infectious bowel disease	Enhanced the gut immune response and decreased the amount of PCV2 in feces and in the ileum	Up-regulated the gene expression of chemokines, IFN-γ, IgA and PIgR and increased the proportion of natural killer cells and the CD19+ lymphocytes in the MLN.
DSM 17938	Kubota et al. (2020)	Newborn Sprague-Dawley rat pups	NEC	Increased the percentage of Foxp3^+^ T cells in the ileum while decreasing the percentage of cells in the MLN; Enhanced anti-inflammatory effect and regulated immune response	Activation of FoxP3^+^ Tregs

IBD, inflammatory bowel disease; HSPs, heat shock proteins; PEG, polyethylene glycol; DSS, dextran sodium sulfate; DCs, dendritic cells; MLNs, mesenteric lymph nodes; FoxP3, forkhead box P3; TNBS, 2,4,6-trinitrobenzenesulfonic acid; Tregs, regulatory T cells; IL, interleukin; NF-κB, nuclearfactor-κB; MUC2, mucin 2; KEGG, kyoto encyclopedia of genes and genome; ILC3s, group 3 innate lymphoid cells; SAA, serum amyloid A; H2R, type 2 histamine receptor; TNF-α, tumor necrosis factor-α; HDC, histidine decarboxylase; AOM, azoxymethane; COX-2, cyclooxygenase-2; IS, immobilization stress; ETEC, enterotoxigenic Escherichia coli; EPEC, enteropathogenic Escherichia coli; CREB, cAMP-response element binding protein; LFCA, lactoferricin-lactoferrampin; IFN-γ, interferon-γ; IgA, immunoglobulin A; PIgR, polymeric Ig receptor; NEC, necrotizing enterocolitis; PCV2, porcine circovirus type 2; HRV, human rotavirus; TH1, helper T cell 1; TH2, helper T cell 2; TGF-β, transforming growth factor-β.

**Table 3:** Therapeutic efficacy and potential mechanisms of *Lactobacillus reuteri* strains in various intestinal diseases in humans.

***Lactobacillus reuteri* strain**	**References**	**Experimental model/participant**	**Disease**	**Effect/outcome**	**Mechanism of action**
RC-14	Jang et al. (2018a)	Human	IBD	The proportion of CD4^+^ CD25 high T cells increased, but TNF-α^+^/IL-12^+^ monocytes and myeloid DC decreased	The underlying mechanisms remain unclear
ATCC 55730	Jang H. M. et al. (2019)	Children(years range 6–18)	IBD	Improved mucosal inflammation and changed mucosal expression levels of some cytokines(IL-10 significantly increased whereas IL-1β, TNFα, and IL-8 significantly decreased)	The underlying mechanisms remain unclear
DSM 17938	Gancarčíková et al. (2019)	Infants younger than 60 days	Colic	Decrease in daily crying time in infants with colic	Activation of FoxP3
SGL01	Hojsak (2019)	Infants aged 3–16 weeks	Colic	Number and duration of crying episodes decreased significantly	The underlying mechanisms remain unclear
FloraActive™ 12246	Pärtty et al. (2018)	Infants aged 4–12 weeks	Colic	Decreased cry and fuss time	The underlying mechanisms remain unclear
ATCC 55730	Roos et al. (2013)	Infants	Colic	Decreased crying time	The underlying mechanisms remain unclear
LR92 DSM 26866	Skórka et al. (2017)	Pregnant women	Colic	Prevented the occurrence and reduce the severity of infantile colic	The underlying mechanisms remain unclear
DSM 17938	Gerasimov et al. (2018)	Children aged 6 months−6 years	CFC	Exhibited significant improvement in defecation frequency	The underlying mechanisms remain unclear
DSM 17938	Pourmirzaiee et al. (2020)	Children with a mean age 9.1 ± 3.8 years	FAP	Reduced the frequency and intensity of abdominal pain episodes	The underlying mechanisms remain unclear

IBD, inflammatory bowel disease; DCs, dendritic cells; TNF-α, tumor necrosis factor-α; FoxP3, forkhead box P3; IL, interleukin; CFC, chronic functional constipation; FAP, functional abdominal pain.

## The basic function of *L. reuteri and* its metabolites

According to incomplete statistics, *L. reuteri* consists of dozens of strains that originated from different samples. In addition, each stain and its unique metabolite may play a distinct role in various intestinal diseases. It is well documented that *L. reuteri* plays multifaceted roles in regulating immune responses, modulating gut microbiota, boosting beneficial metabolites, protecting against oxidative stress, maintaining intestinal barrier (IEB) function and intestinal morphology, and so on (Yi et al., 2018b; Liu et al., 2019; Garg et al., 2020; Singh et al., 2021). In this study, we will list some representative strains to introduce concrete pathogenesis.

It is well established that histamine is beneficial to the intestine, whose synthesis and secretion require l-histidine decarboxylase and a l-histidine/histamine exchanger (Hemarajata et al., 2013; Spinler et al., 2014). The current study identified chloride channel (ClC)-family proton/chloride antiporters as a modulator in the process of histamine production (Hall et al., 2019). *Lactobacillus reuteri* 6475 is special among gut microbes due to it containing a complete chromosomal histidine decarboxylase (*hdc*) gene cluster (genes *hdc*A, *hdc*B, and *hdc*P) and thus having the genetic capacity to convert histidine to histamine (Spinler et al., 2014). *Lactobacillus reuteri*-derived histamine can suppress gut inflammation by activating type 2 histamine receptors (H2R) and restricting pro-inflammatory H1R (Schreiber et al., 2009; Preidis et al., 2012). One of the mechanisms can be attributed to the function of a soluble bacterial enzyme named diacylglycerol kinase (Dgk), secreted by *the L. reuteri* strain, which can diminish Protein Kinase C (PKC) phosphorylation and suppress the H1R signaling pathway in the intestinal epithelium (Ganesh et al., 2018). *Lactobacillus reuteri* 6475 was also related to folate metabolism, which was mediated by dihydrofolate synthase/folylpolyglutamate synthase type 2 (*folC*2). Notably, the *fol*C2 mutant can yield diminished *hdc* gene cluster expression and thus reduced histamine production, hinting at a link between folate and histadine/histamine metabolism (Thomas et al., 2016). Tryptophan (Trp) metabolism is also essential for gut immune homeostasis. The Trp metabolites from *L. reuteri* are known as aryl hydrocarbon receptor (AhR) ligand—indole-3-aldehydes, which can contribute to activating the AhR-IL-22 axis and maintaining intestinal homeostasis (Zelante et al., 2013). Another experiment also disclosed that indole-activated AhR could reprogram intraepithelial CD4^+^ T cells into immunoregulatory T cells to perform regulatory functions (Cervantes-Barragan et al., 2017). Özçam et al. (2019) creatively identified a novel pathway by which *L. reuteri* activates AhR, which is independent of Trp metabolism, known as polyketide synthase (PKS) gene clusters in *L. reuteri* 2010 and R2lc.

As is well-known, the adhesive ability is significant for bacterial function in the intestines of the host. *In vitro* experiments showed that *L. reuteri* has the potential to enhance adhesion in HT-29 cells (Dudík et al., 2020). Given this, Gao et al. (2016) assessed the modulatory effects of two strains of *L. reuteri*—ZJ617 and ZJ615, with different adhesive abilities in *in vivo* experiments. Finally, the authors indicated that both of them can exert anti-inflammatory and anti-oxidative effects, plus metabolism regulation, including glucose and its derivatives, galactose, amino acid metabolism, biosynthesis of antibiotics, and mineral absorption (Gao et al., 2016). Furthermore, the glyceraldehyde-3-phosphate dehydrogenase (GAPDH) protein of *L. reuteri* ZJ617 has been proven to work as an essential adhesion component in binding to the intestinal epithelial cells (Yang et al., 2020). In a harsh context, *L. reuteri* SH23 still retained its adhesive ability with the help of the Mub protein (Xu et al., 2021).

Maintaining the functions of the intestinal epithelium is a key point in protecting against bowel diseases. It was demonstrated that *L. reuteri* D8 has the ability to restore the epithelial damage caused by TNF by activating the Wnt/β-catenin pathway, thus stimulating the proliferation of the intestine, increasing the number of Paneth cells and increasing the expression of antimicrobial peptides (Wu et al., 2020). *Lactobacillus reuteri* 22 also was capable of promoting intestinal stem cell differentiation into goblet cells with increased mucin 2 (Muc-2) expression to ensure the functionality of the intestinal mucosal barrier (Xie et al., 2019). microRNAs (miRNAs) functioned as an agent during the anti-inflammatory course of *L. reuteri* I5007. It was able to maintain intestinal epithelial function by changing the miRNA expression of piglets, especially the PI3K-Akt and MAPK pathways, modulated by different signaling pathways (Wang Q. et al., 2020).

## Association between *L. reuteri* and inflammatory bowel disease

With the acceleration of industrialization and changes in diet, IBD has become an emerging global disease, the incidence of which has risen considerably over the past several decades, both in the Western world and in developing countries (Kaplan, 2015). It is common knowledge that the gut microbiota's roles in the development and course of IBD are significant and enlightening. Given this, probiotics have become a hot topic both in the fundamental research and clinical practice of IBD (Martyniak et al., 2021). Research on *L. reuteri* is thus increasingly important.

### Research on *L. reuteri* in fundamental fields of IBD

Fundamental study of the relationship between IBD and *L. reuteri* can be traced back to 2002. In that study, researchers initially found that a colonized *L. reuteri* strain can prevent the development of colitis in genetically susceptible mice (Guarner et al., 2002). One common feature of IBD is the disruption of the intestinal barrier and, subsequently, an uncontrollable inflammatory signal cascade (Shin and Kim, 2018; Guo F. et al., 2021). Consequently, a collection of studies focused on these two aspects. With the help of the 5(6)-carboxyfluorescein diacetate N-succinimidyl ester (cFDA-SE) labeling technique, Wang et al. (2021) found that endogenous *L. reuteri* R28, isolated from mouse feces, can, significantly ameliorate diarrhea caused by polyethylene glycol (PEG) through its superior colonization in the intestinal environment, regulate the expression of pro-inflammatory factors in mice with colitis induced by PEG + dextran sulfate sodium (DSS), and enhance the intestinal barrier. Marcinkiewicz et al. (2007) used a chronic active colitis animal model to explore the function of *Lactobacillus* strains. Among these, *L. reuteri* was also found to perform anti-inflammatory activities. Macrophages play a key role in the establishment of chronic intestinal inflammation observed in IBD (Heinsbroek and Gordon, 2009). It is well-documented that macrophages have two phenotypes: pro-inflammatory M1-like and anti-inflammatory M2-like. Evidence suggests that GroEL purified from *L. reuteri* can promote macrophage switching to M2-like polarization from the M1-like phenotype to present its anti-inflammatory properties through the Toll-like receptor (TLR) 4 and the non-canonical pathway (Dias et al., 2021). The previous study by Liu et al. (2021) demonstrated that *L. reuteri* can strengthen the intestinal barrier by regulating the expression level of tight junction (TJ) protein and thus protect against colitis in mice. Further, the authors found that *L. reuteri* ATCC PTA 4659 plays an essential role in the anti-inflammatory effect and the related immune reactions by reducing the number of CD11b^+^CD11c^+^ dendritic cells (DCs) and regulating the function of mesenteric lymph nodes (MLNs) (Liu et al., 2022). Similarly, *L. reuteri* 5454 and ATTC PTA 6475 have anti-inflammatory and anti-infectious capacities in 2,4,6-trinitrobenzene sulfonic acid (TNBS)-induced acute colitis in mice by promoting DC maturation, stimulating IL-10 production, and inducing the differentiation of Treg cells and Th17 cells (Hrdý et al., 2020; Engevik et al., 2021).

In addition to having anti-inflammatory effects, *L. reuteri* can also modulate gut microbiota and metabolic disorders in colitis in mice. Wang G. et al. (2020) first isolated *L. reuteri* I5007 from healthy weanling piglets and subsequently examined the effects of *L. reuteri* I5007 in suppressing colonic inflammation, improving colonic microbiota composition, and regulating the metabolic pathways through *in vivo* and *in vitro* models (Hou et al., 2014). In *in vivo* experiments, pretreatment with *L. reuteri* I5007 for 1 week can effectively decelerate DSS-induced weight loss, minimize the reduction in colon length, restore the function of goblet cells, and reduce the production of cytokines. Moreover, it was also beneficial to microbiota and metabolite composition. *In vitro*, the pretreatment of this strain was able to reduce the expression level of IL-1β and TNF-α in HT-29 cells challenged with lipopolysaccharides (LPS) for 4 h. In addition, the authors also revealed that *L. reuteri* treatment improves the DSS-disrupted gut microbial ecology, especially in the colon.

Inflammatory bowel disease is known to be a chronic inflammatory-immune disease, stimulating the exploration of immune-related mechanisms regulated by probiotics, in which *L. reuteri* can play an important role. It is currently widely known that immune checkpoint blockade (ICB) immunotherapy has become a promising cancer treatment (Postow et al., 2015; Khalil et al., 2016). However, it can also have some serious side effects, of which ICB-associated colitis is one of the most common complications (Michot et al., 2016). In the animal experiment of Wang et al. (2019) the authors finally concluded that direct administration of *L. reuteri* ATCC PTA 6475 can facilitate the immunopathology of ICB colitis and, inspiringly, does not exert an impact on the antitumor immunity of ICB by means of significantly decreasing the numbers of mucosal group 3 innate lymphoid cells (ILC3s) and the expression of IL17 and IL23. Nevertheless, the authors did not elaborate on the further mechanism for how *L. reuteri* lowered the numbers of ILC3s.

Some studies suggested that probiotic therapy can attenuate oxidative stress in rats, which is one of the main factors aggravating intestinal injury in IBD (Damiani et al., 2007; Sengül, 2011). In the experiment of Haydn et al., the authors concluded that neither wild-type BR11 nor a CyuC-deficient strain of *L. reuteri* could prevent the development of experimental colitis in rats. Hence, the authors stated that *L. reuteri* BR11 has the ability to reduce the severity of experimental IBD, owing to its unique antioxidant properties and cysteine/cystine-transport system (Turner et al., 1999; Atkins et al., 2012). However, in this study, researchers only explored the relationship between the cystine-uptake system and *L. reuteri* BR11. The further mechanism still requires investigation. *Lactobacillus reuteri* 23272 can also attenuate the effects of stressor exposure on pathogen-induced colitis by downregulating the chemokine CCL2, which was proven to be indispensable in *Citrobacter rodentium*-induced colitis (Mackos et al., 2016).

Anxiety disorder is a common disorder and can progress to depression, which has become a global disease (Baxter et al., 2014; Bandelow and Michaelis, 2015; Weger and Sandi, 2018). A systematic review concluded that patients with IBD have an approximately 20% prevalence rate of anxiety and a 15% prevalence rate of depression (Neuendorf et al., 2016). An ocean of research demonstrated that probiotics have the ability to reduce depression, accompanied by a series of mechanisms, in which *L. reuteri* was found to exert an anti-depressive impact on mice (Davis et al., 2016; Jang et al., 2018a,b). Based on the above, Jang H. M. et al. (2019) investigated the preventive and curative effects of *L. reuteri* NK33, isolated from healthy human feces, on immobilization stress (IS)-induced anxiety/depression and colitis in mice. The findings can be divided into two major parts: (1) *L. reuteri* NK33 exhibited an anti-inflammatory effect by inhibiting the expression of IL-6 and the activation NF-κB pathways in LPS-treated BV-2 cells. Further, *L. reuteri* NK33 could improve intestinal inflammation by restricting the expression of pro-inflammatory cytokines and the infiltration of inflammatory cells and enhancing the abundance of gut microbiota, such as Bacteroidetes, Firmicutes, and Actinobacteria in mice. (2) The treatment with *L. reuteri* NK33 can activate microglial cell infiltration into the hippocampus and induce hippocampal brain-derived neurotrophic factor (BDNF) expression and cAMP-response element binding protein (CREB) phosphorylation in IS-exposed mice as well as LPS-stimulated SH-SY5Y cells by suppressing the activation of the NF-κB pathway and HPA axis, thus suggesting that NK33 alleviated the suppression of NF-κB-mediated BDNF expression in the hippocampus, with the regulation of LPS infiltration into the brain, leading to the attenuation of anxiety and depression. At the same time, this research also uncovered the synergy between *L. reuteri* NK33 and *Bifidobacterium adolescentis* NK98.

Some vital molecules also play indispensable roles in treating IBD using *L. reuteri*, which deserve expounding. Heat shock proteins (HSPs), a type of highly conserved molecular chaperone, work as gatekeepers for intracellular proteins to maintain cell homeostasis (Liu et al., 2014a; Gupta et al., 2017). HSPs, activated by TJ protein, are vital in protecting the gut epithelium against oxidative stress and inflammation, regulating the immune response, and modulating bacterial functions (Liu et al., 2014a, 2022). The research of Liu H-Y. et al. also found that pretreatment with *L. reuteri* ATCC PTA 4659 can enhance the expression of two inducible HSPs, i.e., HSP7 and HSP25, in the distal colon of mice, at mRNA and/or protein levels, by increasing the mean fluorescence intensity (MFI) of HSP70 and HSP25 in both surface mucosa and the crypt as well as expanding their distribution when compared with the control animals. Furthermore, in the colon, the crypt HSP25 expression was negatively correlated with the bacterial load and the Ki67^+^ cell number (Liu et al., 2022).

**Figure 1:**
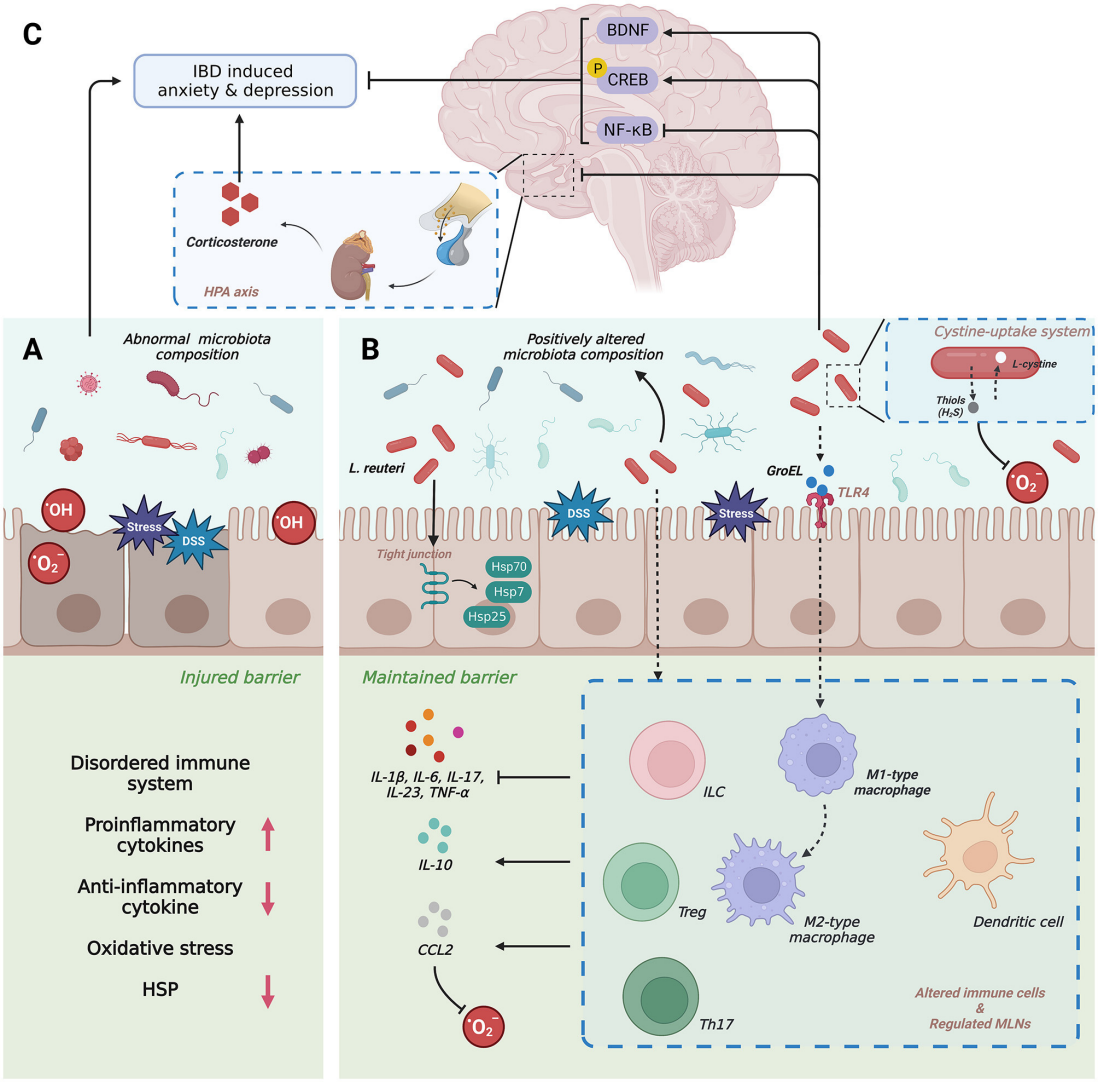
**(A–C)** Schematic diagram depicting the pathogenic role and therapeutic potential of *Lactobacillus reuteri* in IBD. IBD, inflammatory bowel disease; BDNF, brain-derived neurotrophic factor; CREB, cAMP-response element binding protein; DSS, dextran sodium sulphate; HSPs, heat shock proteins; NF-κB, nuclearfactor-κB; IL, interlukin; TNF-α, tumor necrosis factor-κ; CCL, C-C motif chemokine 2; Th, T helper cell; TLR, Toll-like receptors; ILC, innate lymphoid cells; Treg, regulatory T cell; MLN, mesenteric lymph node.

Nevertheless, this study only revealed the expression of HSPs after treatment with *L. reuteri* without elucidating concrete mechanisms. Histamine, the vital molecular in histidine metabolism, played an essential role in the TNBS-induced mouse colitis model. Gao et al. (2017) demonstrated that *hdc*^+^ *L. reuteri* clade II strain 6475, isolated from breast milk, attenuates colonic inflammation through the activation of the histidine decarboxylase (hdc) gene and the histamine H2 receptor (H2R) and supplementation of dietary _L_-histidine (Gao et al., 2015). Regarding this topic, Hemarajata et al. (2013) reported that the *L. reuteri*-specific immunoregulatory (*rsiR*) gene, which originated from gene expression profiles of *L. reuteri* ATCC PTA 6475, is essential for TNF suppression and *hdc* gene expression. Further, the TNBS mouse model lacking the *rsiR* gene fails to exhibit anti-inflammatory effects (Hemarajata et al., 2013). MiR-142a-3p, a type of microRNAs (miRNAs), was found to alleviate colitis by promoting the growth of *L. reuteri* and its metabolite, further affecting the expression of inflammatory genes in intestinal epithelial cells (He et al., 2022) (Figure 1).

### The clinical applications of *L. reuteri* in IBD

Diet therapy has always been a focus in the treatment of IBD. In the study of Kim et al. (2020) the conclusion indicated that mango intake significantly reduced biomarkers of inflammation and modulated the intestinal microbiota, which significantly increased the abundance of *L. reuteri* (Kim et al., 2020). The experiment by Sun et al. (2018) also supported this idea. The authors found that the space flight–induced mutant *L. reuteri* F-9-35 has excellent potential for the prevention of UC as a dietary supplement compared with that of the wild type and milk alone (Sun et al., 2018). With the maturity of the probiotics industry, probiotics have also been added to the daily diet. For instance, one prospective study used *L. reuteri* RC-14–supplemented yogurt as atherapy and finally confirmed its anti-inflammatory effect on patients with IBD (Lorea Baroja et al., 2007). As for children with active distal UC, a randomized clinical trial showed the effectiveness of *L. reuteri* ATCC 55730 in improving mucosal inflammation and reducing the expression level of some iconic cytokines (Oliva et al., 2012).

Nevertheless, the number of clinical research studies on *L. reuteri* in IBD is less than that of fundamental studies. We attribute this phenomenon to the speculation that *L. reuteri* is composed of multiple stains. Each stain may have different functions that must be fully investigated in fundamental experiments.

## Colorectal cancer

Colorectal cancer (CRC) ranks as the third most common type of cancer and the fourth leading cause of cancer-related deaths globally (Weitz et al., 2005). It is well-known that several risk factors have been associated with the occurrence and development of CRC, such as inflammatory bowel disease (IBD), age, and genetic, and environmental factors (Keller et al., 2019; Thanikachalam and Khan, 2019). Probiotic therapy has become a hotspot treatment for CRC (Ambalam et al., 2016; Fong et al., 2020). Researchers demonstrated that *L. reuteri* ATCC PTA 6475 had the ability to reduce the number and size of colon tumors, with the mechanism that administration of *hdc*^+^ *L. reuteri* led to *hdc* gene expression and histamine production in the gut to suppress chronic intestinal inflammation and colorectal tumorigenesis (Gao et al., 2017). In addition, the interaction between Sirt3 and *L. reuteri* was proven to be crucial in gut tumorigenesis (Zhang et al., 2018). The research conducted by Bell et al. (2022) acquired some convincing results. *In vitro*, the authors found fecal metabolites from wild-type mice and normal humans. Both can inhibit the proliferation of CRC cell lines but not repress the noncancerous cell line NCM460. Metabolomics finally identified reuterin as the most inhibitory compound. According to a previous study, we learned that reuterin was an antimicrobial produced by *L. reuteri*—an intermediate in glycerol metabolism to 1,3-Propanediol (Martín-Cabrejas et al., 2017; Asare et al., 2020; Zhang et al., 2020). In addition, reuterin at a dose of 25 μM could inhibit the growth of CRC cell lines (HCT116, SW480, RKO, and DLD1), but a higher concentration of reuterin (100 μM) had no effect on normal colon epithelial cells. *In vivo*, the authors concluded that *L. reuteri* was reduced in tumors compared with normal tissues.

Moreover, the authors confirmed this result by using public datasets and patient tissue samples. Based on metabolomics and gene-enriched analysis, it was found that *L. reuteri* growth was suppressed by the homocysteine degradative metabolites hydrogen sulfide and cystathionine. *Lactobacillus reuteri* growth could not be altered by supplementing with the antioxidant glutathione ethyl ester, which indicated that the oxidative stress pathway was not specific to *L. reuteri*. In a bid to explore the concrete mechanism, the authors treated an intestinal cell line with 100 μM reuterin for 24 h. With the help of metabolomics, transcriptomics, and proteomics, the authors observed upregulation of the nuclear factor erythroid 2–related factor 2 (NRF2), which played an essential role in the oxidative stress response. The most enriched pathway focuses on glutathione and glutamate metabolism. Quantification of oxidized L-glutathione confirmed the role reuterin played in oxidative stress. With the subsequent observation that acetylcysteine (NAC) inhibited the induction of NRF2-dependent oxidative stress genes, it was strongly confirmed that reuterin directly controls the redox balance of a cell in a glutathione (GSH)-dependent manner. Creatively, researchers found that sodium sulfide protected cells against reuterin-induced growth inhibition, along with the result that reuterin selectively bound to cysteine residues in numerous biological replicates, indicating the significantly different cysteine proteomics profile of reuterin. The NAD pathway was also involved in the oxidative stress process. Using RNA-sequencing analysis, the puromycin incorporation assay (SUnSET), and a cell inhibition experiment, the authors finally identified the inhibiting ribosomal assembly as an essential cytotoxic pathway of reuterin, in which YEATS2 target genes were found to be downregulated after treatment with reuterin.

**Figure 2:**
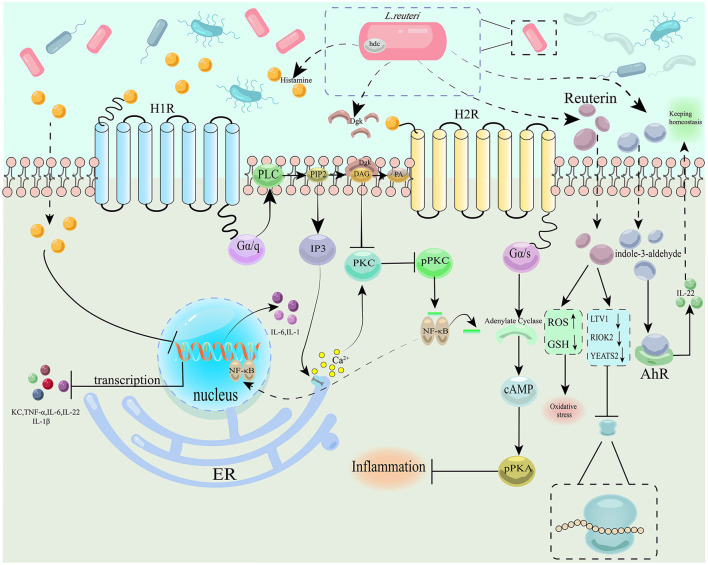
Schematic diagram depicting the pathogenic role and therapeutic potential of *Lactobacillus reuteri* in CRC. H1R, type 1 histamine receptor; H2R, type 2 histamine receptor; hdc, histidine decarboxylase; PLC, phospholipase C; PIP2, phosphatidylinositol 2; Dgk, diacylglycerol kinase; DAG, diacylglycerol; PA, phosphatidic acid; IP3, inositol triphosphate; PKC, protein kinase C; pPKC, phosphorylated protein kinase C; ER, endoplasmic reticulum; AhR, aryl hydrocarbon receptor; PKA, protein kinase A; pPKA, phosphorylated protein kinase A; KC, keratinocyte chemoattractant; ROS, reactive oxygen species; GSH, glutathione; LTV1, LTV1 ribosome biogenesis factor; RIOK2, right open reading frame kinase 2; YEATS2, YEATS domain-containing protein 2.

Taken together, reuterin was capable of repressing colorectal cancer growth *in vivo*. This excellent study opened new avenues for researchers. Christina Watschinger and Alexander R. Moschen expressed their distinct opinions on this comprehensive research (Watschinger and Moschen, 2022). The authors further desired to determine how reuterin's selectivity is mediated and the mechanism by which reuterin accumulates in tumor cells outside the gut. All of these outstanding findings can contribute to enhancing the transformation from basic research to clinical application. Along with the experiments on living bacteria and their secrets, Kim et al. (2022) creatively reported the joint function of heat-killed *L. reuteri* MG5346 and *L. casei* MG4584 in human CRC. The authors ultimately demonstrated that both of these strains could play an antitumor role through the caspase-9-dependent apoptosis pathway (Figure 2).

## Infection-associated intestinal diseases

Enterotoxigenic *Escherichia coli* (ETEC) is a leading cause of infectious diarrhea in humans and animals. In the study of Xie et al., the authors creatively developed a new type of *L. reuteri—*a bovine lactoferricin-lactoferrampin (LFCA)-encoding *L. reuteri CO21* (LR-LFCA) and finally demonstrated that LR-LFCA can function in the following three aspects in a newborn ETEC-infected piglet intestine model: (1) it could enhance gut immune responses by improving intestinal barrier function and gut microbiota composition, (2) it was able to protect the gut from oxidative stress by activating the NRF2/HO-1 pathway, and (3) it had the ability to inhibit the NF-κB pathway to perform its anti-inflammatory effect (Xie et al., 2021). Further, Tkáčiková et al. (2020) found that the pretreatment of *L. reuteri* L26 Biocenol (CCM 8616)-derived bacterial exopolysaccharides (EPSs) can attenuate the overexpression of the genes induced by ETEC infection to suppress inflammatory responses. Human-derived *L. reuteri* strains (ATCC PTA 6475, DSM 17938, and 1563F), a rat strain (R2LC), and piglet-derived *L. reuteri* (HCM2 and LR1) were also able to reduce the detrimental effect of ETEC (Karimi et al., 2018; Wang et al., 2018; Yi et al., 2018a). Reuteran and Levan, two metabolites produced by *L. reuteri* TMW1.656 and *L. reuteri* LTH5794, respectively, can also reduce the colonization of weanling piglets by ETEC (Yang et al., 2015). As for enteropathogenic *E. coli* (EPEC), there was evidence suggesting that *L. reuteri* ATCC PTA 6475 and ATCC 53608 significantly inhibited EPEC by targeting either the epithelium or the mucus layer, depending on the strain's specialty (Walsham et al., 2016). Notably, *L. reuteri* ATCC PTA 6475 was also effective in suppressing enterohemorrhagic *E. coli* (EHEC) (Eaton et al., 2011). With respect to *Salmonella* infections, it was demonstrated that *L. reuteri* Lb11, isolated from the chicken intestinal tract, can effectively prevent the formation of an efflux pump, inhibiting the production of multidrug-resistant *Salmonella* enteritidis in eggs (Hai and Huang, 2021). *Lactobacillus reuteri* KUB-AC5 can also protect against *Salmonella* infection in chickens (Nakphaichit et al., 2019). Jiang P. et al. (2019) revealed that *L. reuteri* ATCC 55730 can prevent mice from acquiring *Salmonella* Typhimurium by activating macrophages to produce nitric oxide. A combination of a phage cocktail and *L. reuteri* was able to ameliorate mouse colitis caused by *S*. Typhimurium by improving the intestinal barrier and colonic pathological damage, in which the metabolites of *L. reuteri-*acetate and reuterin played important roles (Eaton et al., 2011). Remarkably, glycerol supplementation had the ability to enhance *L. reuteri* ATCC PTA 6475 's protective effect against *S*. Typhimurium colonization (De Weirdt et al., 2012). *Lactobacillus reuteri* CCM 8617 and SLZX19-12 were also shown to exert vital impacts on *S*. Typhimurium (Gancarčíková et al., 2019; Wu et al., 2022). In regard to virus infection, some investigations have already indicated that *L. reuteri* ATCC 23272 functions as a significant modulator in gnotobiotic pigs infected with human rotavirus, and *L. reuteri* L26 Biocenol™ plays an essential role in protecting against porcine circovirus type 2 (Azevedo et al., 2012; Karaffová et al., 2017).

## Pediatric intestinal diseases

### Functional intestinal disorders

At present, the majority of the research for *L. reuteri* on functional intestinal disorders focuses on clinical research conducted on children and infants. Infantile colic, functional constipation (FC), functional abdominal pain (FAP), and irritable bowel syndrome (IBS) are the most common functional gastrointestinal disorders in children (Hojsak, 2019). In these fields, probiotics have proven to be promising therapeutic options (Pärtty et al., 2018). In 2013, a randomized DBPC trial showed no difference in microbiota between colicky infants with or without treatment using *L. reuteri* DSM 17938 (Roos et al., 2013). A systematic review also summarized the effects of *L. reuteri* ATCC 55730 and *L. reuteri* DSM 17938, concluding that none affected infantile colic relief (Skórka et al., 2017). However, several studies indicated that treatment with *L. reuteri* DSM 17938 can relieve infantile colic (Savino et al., 2018a,b, 2019; Turco et al., 2021). A combination containing heat-killed *L. reuteri* SGL01 and *Bifidobacterium brevis* SGB01 had better curative effects in infantile colic than ordinary dietary supplements (Vandenplas et al., 2017). *Lactobacillus reuteri* (FloraActive™) 12246, *L. reuteri* (American Type Culture Collection Strain 55730), and LR92 DSM 26866 all came into play in infantile colic (Savino et al., 2007; Gerasimov et al., 2018; Pourmirzaiee et al., 2020). Over 90% of child-associated constipation can be classified as FC, and some meaningful studies have been conducted in this area (Tambucci et al., 2018). Some reviews summarized the effectiveness of *L. reuteri* DSM 17938 in infants and children, with the conclusion that it is not recommended to use *L. reuteri* DSM 17938 routinely in the management of infants with constipation (Urbańska and Szajewska, 2014; Wegh et al., 2018). Similarly, some randomized controlled trials also found that *L. reuteri* DSM 17938 is not beneficial for the treatment of FC in children (Jadrešin et al., 2018; Wegner et al., 2018). Conversely, Kubota et al. (2020) noted a remarkable improvement in the defecation frequency with *L. reuteri* DSM 17938 and Magnesium Oxide in FC. Current research on FAP in children focuses on *L. reuteri* DSM 17938. Authors have found that *L. reuteri* DSM 17938 effectively alleviates pain and restores normal activities in children with FAP (Weizman et al., 2016; Maragkoudaki et al., 2017; Jadrešin et al., 2020; Trivić et al., 2021). With respect to IBS, the corresponding studies were marginal, and their findings all indicated that *L. reuteri* DSM 17938 is unable to improve the symptoms of IBS (Niv et al., 2005; Jadrešin et al., 2020). Another functional disorder in children, diarrhea, can also be relieved by *L. reuteri* DSM 17938 (Gutierrez-Castrellon et al., 2014). From the above interpretations, we can see that *L. reuteri* does not = have effective therapeutic results. Our point of view can be divided into two aspects: (1) this can be attributed to the limitation of sample size because nearly all studies' sample size was < 100 cases and (2) current trials are mostly centered on *L. reuteri* DSM 17938 and perhaps other strains would have some effects we still do not know. Thus, more trials need to be carried out in these areas.

### Necrotizing enterocolitis

Necrotizing enterocolitis (NEC) is a common intestinal disease that occurs in premature infants and is the leading cause of short bowel syndrome in neonates (Neu and Walker, 2011). It is well documented that *L. reuteri* has become an effective treatment for this disease. *Lactobacillus reuteri* DSM 17938 can improve survival and reduce the incidence and severity of NEC by modulating the immune response and the induction and migration of Foxp3^+^ regulatory T cells (Tregs) (Liu et al., 2013). Further, this research team found that the anti-inflammatory effect of *L. reuteri* DSM 17938 on NEC relied on differential modulation of effector memory T cells and Foxp3^+^Tregs (Liu et al., 2014b). In 2018, the authors also discovered that TLR2 could play a part in alleviating NEC by means of activating DC (Hoang et al., 2018). Based on previous research results, the authors conducted their experiment on newborn mice by feeding experimental animals *L. reuteri* DSM 17938, concluding that oral administration of this probiotic can increase levels of tryptophan metabolites and purine nucleoside adenosine and can be beneficial to general health (Liu et al., 2019). Probiotic persistence is a major topic in probiotic therapy. Given this, Olson et al. (2018) have fully used biofilm's function to enhance the persistence of *L. reuteri* in the protection against NEC. Similarly, Al-Hadidi et al. (2021) also developed a new formulation of enterally delivered probiotics to improve probiotic survival through biofilm formation. Shelby et al. (2022) showed that, compared with the planktonic state of *L. reuteri*, its biofilm state significantly decreased the incidence of NEC through antibacterial and anti-inflammatory effects.

## Conclusions and future perspectives

A growing number of studies showed that intestinal diseases can cause mounting healthcare bills and economic burdens. It is well-documented that gut microbiota plays an increasingly essential role in the treatment and prognosis of gut diseases. *Lactobacillus reuteri* is a common and well-studied microbe. Extensive investigations have been conducted in this area. However, we still have numerous unanswered questions. As a gut symbiont, *L. reuteri* can be colonized in the intestine of humans, rodents, pigs, and chickens and can perform multiple actions, including regulating immune responses, modulating gut microbiota, boosting beneficial metabolites, protecting against oxidative stress, maintaining intestinal barrier (IEB) function and intestinal morphology, and so on (Yi et al., 2018b; Liu et al., 2019; Garg et al., 2020; Singh et al., 2021). In this review, we first elucidated the basic function of *L. reuteri* and its related metabolites. Next, we systematically interpreted its function in different intestinal diseases, such as inflammatory bowel disease, colorectal cancer, infection-associated bowel disease, and pediatric intestinal disorders. We also emphasized some vital molecules in association with the underlying mechanisms. Cumulatively, *L. reuteri* is potentially beneficial to intestinal diseases, which should be further investigated in a bid to obtain better clinical application and therapeutic effects.

Although an increasing number of research studies on *L. reuteri* are well-studied by current researchers, there are still some key issues that are in doubt. First, there is a substantial gap between basic research and clinical applications based on the present literature data, probably owing to the unspecific mechanisms and doubtful safety of this microbe. Safety is an important issue for the wide application of probiotics. Conducting standardized safety assessments and finding effective methods to control the side effects of probiotics may be the future research direction. In addition, the paradoxical results of clinical research also restrict the development of *L. reuteri*, which may be attributed to the fact that *L. reuteri* has many distinct strains, and each strain may have its own unique function, for better or worse.

We need to fully evaluate the clinical effect of each strain and the mechanism underlying it. These issues may be addressed with the improvement of industrialized probiotics and experimental techniques. At last, the development of multiple omics analyses, especially metabonomics, allows us to investigate the functions of *L. reuteri's* metabolites, which may help us thoroughly investigate this field. Based on this, researchers can develop metabolites-targeted probiotic products, contributing to the refinement management of the probiotic industry.

## Author contributions

ZY, JC, and YL: writing—original draft and visualization. QM, HL, QY, WS, and XR: conceptualization. XC: conceptualization and writing—review, editing, and supervision. All authors contributed to the article and approved the submitted version.
